# Unintended Consequences of not Specifying Exclusionary Illnesses for Systemic Exertion Intolerance Disease

**DOI:** 10.3390/diagnostics5020272

**Published:** 2015-06-23

**Authors:** Leonard A. Jason, Madison Sunnquist, Bobby Kot, Abigail Brown

**Affiliations:** Center for Community Research, DePaul University, Chicago, IL 60614, USA; E-Mails: MSUNNQUI@depaul.edu (M.S.); BKOT@depaul.edu (B.K.); ABROWN57@depaul.edu (A.B.)

**Keywords:** Myalgic Encephalomyelitis, chronic fatigue syndrome, systemic exertion intolerance disease, case definitions

## Abstract

The Institute of Medicine recently proposed a new case definition for chronic fatigue syndrome (CFS), as well as a new name, Systemic Exertion Intolerance Disease (SEID). Contrary to the Fukuda *et al.*’s CFS case definition, there are few exclusionary illnesses specified for this new SEID case definition. The current study explored this decision regarding exclusionary illnesses using the SEID criteria with four distinct data sets involving patients who had been identified as having CFS, as well as healthy controls, community controls, and other illness groups. The findings indicate that many individuals from major depressive disorder illness groups as well as other medical illnesses were categorized as having SEID. The past CFS Fukuda *et al.* prevalence rate in a community based sample of 0.42 increased by 2.8 times with the new SEID criteria. The consequences for this broadening of the case definition are discussed.

## 1. Introduction

The Institute of Medicine (IOM) [1] recently proposed a new case definition, which was intended to replace the Fukuda *et al.* [2] chronic fatigue syndrome (CFS) criteria, the most widely used case definition for the past twenty years. The Fukuda *et al*. criteria [2] require four symptoms out of a possible eight, but it is possible that some individuals who meet these diagnostic criteria do not have core symptoms of the illness, such as post-exertional malaise. With the Fukuda *et al.* case definition [2], there are about a million people estimated to have this illness in the US [3]. In reaction to limitations in the Fukuda *et al.* case definition [2], the Canadian Clinical Criteria Myalgic Encephalomyelitis/chronic fatigue syndrome (ME/CFS) [4] was developed, and it specified core symptoms, including post-exertional malaise, impairment of memory and concentration, unrefreshing sleep, arthralgia and/or myalgia; and several autonomic, neuroendocrine, and immune manifestations. Still later, the International Consensus Criteria for Myalgic Encephalomyelitis (ME-ICC) criteria [5] were developed, and these criteria specified eight symptoms within four domains: Post-Exertional Neuroimmune Exhaustion; Neurological Impairments; Immune, Gastro-intestinal, and Genitourinary Impairments; and Energy Production/Transportation Impairments. Others have tried to develop more empiric-based methods [6]. Each of these case definitions excluded a variety of medical or psychiatric illnesses that might be the cause of the symptoms.

Recently, the IOM [1] issued a report that proposed a new name (Systemic Exertion Intolerance Disease, SEID) and case definition that included the following four symptoms: substantial reduction or impairment in the ability to engage in pre-illness levels of occupational, educational, social or personal activities; post-exertional malaise; unrefreshing sleep; and at least one of the two following symptoms: cognitive impairment or orthostatic intolerance. Whereas the Fukuda *et al.* [2] CFS criteria, the ME/CFS Canadian criteria [4], and the ME-ICC criteria [5] excluded other medical and psychiatric conditions that might have produced the fatigue, the new SEID criteria [1] had a different position regarding exclusionary conditions. The IOM [1] (p. 186) document defining SEID stated: “Over the years, case definitions of ME/CFS have differed significantly in their classification of exclusionary conditions and comorbidities. As a result, a number of disorders, such as morbid obesity and an array of psychiatric disorders, are listed as exclusionary in one definition and as comorbid in another, despite the lack of scientific evidence that being affected by such disorders precludes having ME/CFS. Indeed, it has become increasingly clear that many patients with ME/CFS have other disorders as well…Some of these other disorders may develop as part of the spectrum of ME/CFS or in response to the burdens of this disorder.” In addition, within the IOM [1] (p. 185) SEID document, it states that a detailed history and comprehensive physical examination should be used “to determine a differential diagnosis and, where clinically indicated, to exclude other disorders that could cause the patient’s symptoms, as well as to identify any comorbid conditions”. More details on exclusions are provided within the IOM’s SEID Report Guide for Clinicians [7] (p. 4), where it states: “The presence of other illnesses should not preclude patients from receiving a diagnosis of ME/CFS (SEID) except in the unlikely event that all symptoms can be accounted for by these other illnesses.” The word “unlikely” conveys the impression that most other illnesses would be considered comorbid and not exclusionary as they probably would not account for the unique SEID symptoms.

The problem for diagnosticians in interpreting these guidelines is that the core IOM symptoms are not unique to SEID, as other illnesses have comparable symptoms (e.g., cancer, Hashimoto’s, lupus, chronic heart failure, multiple sclerosis, *etc.*). Thus, according to the above IOM guidelines, if these illnesses account for the SEID symptoms, then it is another illness and not SEID. Therefore, many illnesses are now considered a comorbid condition with SEID. However, trying to determine whether an illness is exclusionary *vs.* comorbid is a challenging diagnostic task. The IOM [1] (p. 187) provides the following example that illustrates this complexity: “The committee recognizes that diagnosis and treatment of comorbid conditions is necessary when caring for patients. For example, a patient with ME/CFS with a prominent history of snoring and sleep apnea may have polysomnography diagnostic of sleep apnea. Treatment with continuous positive airway pressure could improve the patient’s overall condition but not resolve all the symptoms of ME/CFS, signifying that in this individual, obstructive sleep apnea is a comorbid condition rather than the cause of the patient’s ME/CFS symptoms.” This suggests that if treatment resolved all the SEID symptoms, then the patient had another illness (in the case above, obstructive sleep apnea); however, if the treatment does not resolve the issues, than the condition is comorbid with SEID. In other words, the ability to determine if an illness is exclusionary rests on its successful treatment, and clearly, many chronic illnesses do not have treatments that cure or alleviate all symptoms.

In addition, Ze-dog [8] pointed out that this new SEID definition lacks exclusion criteria, and as a consequence, it is easier for a person with a primary psychiatric diagnosis to be labeled as having SEID. Verrillo [9] also commented on these exclusionary SEID ambiguities, and then suggested that because major depression is not exclusionary, patients with a primary psychiatric disorder might be included in the SEID classification. These publications were only commentaries and did not provide data, so it is still unclear whether the SEID case definition [1] could inappropriately include cases of purely affective disorders, such as Major Depressive Disorder (MDD). It is also unclear whether SEID is more common within other autoimmune illnesses such as MS and Lupus. The present study evaluated whether the SEID case definition distinguished between persons with MDD, and other illnesses, using archival data that were available. We used data from four distinct studies, each with different case ascertainment methods, so we could begin to determine how the new SEID criteria might affect a variety of samples representing tertiary care settings, community based settings, as well as more patient self-diagnosed samples. We hypothesized that individuals with a number of formerly exclusionary illnesses would meet the SEID case definition, thus possibly increasing the prevalence rate of this illness.

## 2. Methods

### 2.1. Study 1

#### 2.1.1. Procedure

In the first study, a CFS screening questionnaire had a combination of existing and new measures including: (1) several demographic related items; (2) The Fatigue Scale [10]; and (3) a list of symptoms associated with CFS. Interviewees were asked a series of questions that assessed whether or not they had a number of symptoms commonly experienced by people with CFS. The symptoms needed to be experienced for 6 or more months. The questions were asked by interviewers (for more details of this study, see [11]).

#### 2.1.2. Participants

A total of 60 individuals (15 with CFS, 15 Controls, 15 with Multiple Sclerosis (MS), and 15 with Lupus) were recruited from the greater Chicago area for the present study. Fifteen of the participants were diagnosed by a physician in Chicago with experience in diagnosing and treating CFS. Each of these participants met the Fukuda *et al.* [2] definition of CFS. To be diagnosed with the CFS Fukuda *et al.* [2] criteria, participants had to experience persistent or relapsing fatigue for a period of six or more months concurrent with at least four of eight somatic symptoms that do not predate the fatigue. These symptoms are: sore throat, lymph node pain, muscle pain, joint pain, post-exertional malaise, headaches of a new or different type, memory and concentration difficulties, and unrefreshing sleep. Participants also needed to experience substantial reductions in occupational, educational or personal activities as a result of the illness and must not have any exclusionary medical or psychiatric illnesses.

Fifteen healthy control participants had not been diagnosed with CFS or any other illness that could cause significant fatigue. These participants had also been seen by a physician, and no illnesses that could cause fatigue were found (e.g., unresolved cases of hepatitis C virus infection, untreated hypothyroidism). In addition, fifteen participants with a diagnosis of Multiple Sclerosis (MS) were recruited from self-help groups in the Chicago area. Each of these participants met Poser *et al.*’s [12] criteria for definite MS. Participants with other chronic medical conditions in addition to MS were excluded. Finally, fifteen participants with a diagnosis of Systemic Lupus Erythematosus (SLE) were recruited from self-help groups in the Chicago area. The participants with Lupus had to meet the SLE criteria as defined by the American Rheumatology Association [13]. There were no significant differences between groups with respect to race, age, education, marital status, and occupation. However, there were significantly fewer women in the healthy control group as compared to the other groups, and significantly more people were on disability in the CFS and MS group compared to the healthy control group.

#### 2.1.3. SEID Diagnosis

To meet the SEID criteria [1] within this sample, a patient needed to have 6 or more months of illness. To meet the substantial reduction from previous levels of functioning criteria, a patient would have needed to have 6 or more months of substantial reduction in functioning. To meet the post-exertional malaise criteria, a patient would need to have indicated presence of at least 1 of our two post-exertional malaise symptoms: sickness/fatigue for >24 h after exercising or experiencing high levels of fatigue after everyday activity. To meet the unrefreshing sleep criteria, a patient would need to indicate unrefreshed sleep that is more frequent than their pre-illness levels. In order to meet the cognitive impairment criteria, a patient would need at least one of the following cognitive items: difficulty concentrating, difficulty finding the right word to say, difficulty with memory, or difficulty remembering things. Due to a lack of items that tapped into orthostatic intolerance criteria, patients would instead need to meet the cognitive impairment criteria to qualify for the SEID criteria. In another study, we found the option to have orthostatic intolerance instead of cognitive impairment typically enabled only approximately 2% more participants to meet SEID criteria [14].

#### 2.1.4. Results

As indicated in Table 1, 100% (*n* = 15) of those in the CFS group met the SEID criteria, whereas 47% (*n* = 7) in the Lupus group, 33% (*n* = 5) in the MS group, and 0% in the control group met the SEID criteria. In an effort to compare this new SEID case definition to the older Fukuda *et al.* [2] criteria, we computed the sensitivity and specificity. The SEID criteria evidenced a sensitivity of 1.0 (indicating that 100% of participants with CFS were correctly identified by the SEID criteria) and a specificity of 0.73 (indicating that 27% of participants without CFS were classified as meeting the SEID criteria).

**Table 1:** CFS, MS, Lupus, and Control Sample *n* = 60.

Diagnosis	Percent Who Qualify for SEID
CFS (*n* = 15)	100% (*n* = 15)
MS (*n* = 15)	33% (*n* = 5)
Lupus (*n* = 15)	47% (*n* = 7)
Control (*n* = 15)	0% (*n* = 0)

### 2.2. Study 2

#### 2.2.1. Procedure

In the second study, participants were screened by a trained interviewer to determine if they met the inclusion and exclusion criteria for CFS, MDD, or healthy controls (for more details, see [15]). As part of this screening process, all participants were administered the SCID-IV [16] to assess for psychiatric conditions. Participants who met criteria for participation were asked to complete a battery of questionnaires that measured demographics, social, emotional, and physical functioning, activity level, depression, and a comprehensive list of physical, cognitive, and emotional symptoms. Participants were asked to provide data for fatigue and the 8 diagnostic symptoms specified by the Fukuda *et al.* [2] case definition. They were asked to report if each symptom had been present for 6 months or longer, began before the onset of their fatigue or health problems, how often it was experienced, and rated the intensity of each symptom on the same scale of 0 to 100. A prior study by King and Jason [17] found that the CFS group against the MDD and control group had significant differences for the following items rated on severity: 4 symptoms in the fatigue/weakness group (fatigue, post-exertional malaise, muscle weakness, need to nap each day), 3 symptoms in the neuropsychological category (frequently losing train of thought, difficulty finding the right word, confusion/disorientation), 4 symptoms in the infectious category (sore throat, tender lymph nodes, hot and cold spells, feeling chilled/shivery), 3 symptoms in the rheumatologic category (muscle pain, pain in multiple joints without swelling, night sweats), 1 symptom in the cardiopulmonary category (shortness of breath), 1 symptom in the neurological category (blurred vision) and unrefreshing sleep. Therefore, these items were also used in the present study.

The Structured Clinical Interview for the DSM-IV (SCID) is a valid and reliable semi-structured interview guide that closely resembles a traditional psychiatric interview [16]. The SCID is designed to identify current, past, and lifetime (chronic or recurring, current and past) diagnoses for a majority of DSM-IV, Axis I psychiatric disorders. The SCID is commonly administered during a single session lasting 45 min to an hour. Diagnostic decisions generated by the SCID are based on all possible sources of historical, symptomatic, and behavioral information. The SCID begins with a semi-structured interview portion designed to yield a tentative diagnosis. The tentative diagnosis is then systematically assessed during the structured portion of the interview through the use of embedded questions that conform to the exact, Axis I criteria set forth by the DSM-IV.

The SF-36 is 36-item instrument that is comprised of multi-item scales that assess physical functioning, role limitations, social functioning, bodily pain, general mental health, vitality, and general health perceptions. Higher scores indicate better health, lower disability, or less impact of health on functioning. Reliability and validity studies have demonstrated that the 36-item version of the SF-36 has high reliability and validity in a wide variety of patient populations [18].

#### 2.2.2. Participants

A total of 45 individuals (15 with CFS, 15 with MDD, and 15 healthy controls) were recruited from the greater Chicago area [15]. Fifteen participants with CFS were solicited to participate in the present study. Participants were drawn from two sources, a local CFS support group in Chicago and previous research studies conducted at DePaul University. Participants were required to have been diagnosed with CFS, using Fukuda *et al.*’s [2] diagnostic criteria, by a Board-certified physician and were required to have a current (active) case of CFS. All participants had been seen by their physician in the past year. Individuals who reported having uncontrolled or untreated medical illnesses (e.g., untreated anemia) were excluded. All participants were screened with the SCID-IV to ensure that they did not have any exclusionary psychiatric illnesses as stipulated by the Fukuda *et al.* [2] case definition.

Fifteen participants with a diagnosis of MDD were solicited from a local chapter of the National Depressive and Manic Depressive support group in Chicago. Participants were required to have been diagnosed with major depression by a licensed psychologist or psychiatrist. All participants were screened with the SCID-IV to ensure that they met criteria for a current (active) case of major depression and did not have any other current psychiatric illnesses. Individuals who had other current psychiatric conditions in addition to major depression were excluded. Individuals who reported having uncontrolled or untreated medical illnesses (e.g., anemia, diabetes) were also excluded. In the MDD group, all 15 (100%) participants met DSM-IV diagnostic criteria for MDD. None of the participants in the MDD group met criteria for MDD with catatonic, melancholic, psychotic, or atypical features. Participants in the MDD group did not meet criteria for any other Axis I disorders.

Finally, fifteen healthy control participants were solicited from the greater Chicago area. Individuals who did not have any medical illnesses or who did not have any uncontrolled or untreated illnesses (e.g., anemia, diabetes) were allowed to participate. All participants were screened with the SCID-IV to ensure that they did not have any current psychiatric illnesses. Individuals with current psychiatric conditions were excluded. Sociodemographic data were compared across the three groups, and there were no significant differences with respect to gender, race, age, SES, education, marital status, occupation, work status, and additional roles [15].

#### 2.2.3. SEID Diagnosis

To meet the SEID criteria [1] within this sample, a patient would need to have 6 or more months of fatigue. Because the SEID criteria do not indicate how to assess substantial reductions, we used criteria that has been published in prior studies with specified cut off points [6,14]. To meet substantial reduction from previous levels of functioning criteria, a patient needed to meet 2 of the following 3 SF-36 criteria: role physical <50, social functioning <62.5, or vitality <35. To meet the post-exertional malaise criteria, a patient needed to have 6 or more months of post-exertional malaise. To meet the unrefreshing sleep criteria, a patient needed to have 6 or more months of unrefreshing sleep. To meet the SEID criteria, the individual needed to have either a cognitive impairment or orthostatic intolerance symptom. In order to meet the cognitive impairment criteria, a patient would need at least one of the following cognitive items: impaired memory present for 6 months or longer, slowness of thought, absent mindedness or forgetfulness, or difficulty focusing. To meet the orthostatic intolerance criteria, a patient would need presence of at least one of the following items: dizziness, wobbling feet when getting up.

#### 2.2.4. Results

As indicated in Table 2, 93% (*n* = 14) of those in the CFS group, 27% (*n* = 4) in the MDD group, and 0% in the control group met SEID criteria. In an effort to compare this new SEID case definition to the older Fukuda *et al.* [2] criteria, we computed the sensitivity and specificity. These criteria resulted in a sensitivity of 0.93 and a specificity of 0.86.

**Table 2:** CFS *vs.* MDD Database *n* = 45.

Diagnosis	Percent Who Qualify for SEID
CFS (*n* = 15)	93% (*n* = 14)
MDD (*n* = 15)	27% (*n* = 4)
Control (*n* = 15)	0% (*n* = 0)

### 2.3. Study 3

#### 2.3.1. Procedure

The data were derived from a larger community-based study of CFS that was carried out in three stages [3]. Stage 1 entailed a cross-sectional screening telephone survey of a random sample of 28,673 households, with 18,675 adults completing the screening interview (65.1% completion rate). Of these participants, 780 (4.2%) of the respondents had six or more months of fatigue. Stage 2 involved a structured psychiatric interview for a sample of those respondents from Stage 1 who screened positive for a CFS-like syndrome based on the Fukuda *et al.* [2] criteria, as well as a screen negative control sample. In Stage 3, a physician conducted a detailed medical examination to rule out exclusionary medical conditions. All patients underwent detailed reviews of their medical history and a thorough physical and neurological examination to detect evidence of diffuse adenopathy, hepatosplenomegaly, synovitis, neuropathy, myopathy, cardiac or pulmonary dysfunction. All had routine blood tests performed including complete blood count, chemistry screen (including glucose, electrolytes, calcium, magnesium, liver function tests and renal function tests), sedimentation rate, rheumatoid factor, Antinuclear Antibody, triiodothyronine, thyroxine, thyroid-stimulating hormone, Creatine phosphokinase, human immunodeficiency virus, hepatitis screen, B12, red blood cell folate and serum carnitine determinations. All patients had a urinalysis performed.

#### 2.3.2. Participants

According to the Phase 1 screen, of the 18,675 interviewees, 16,453 (88%) had no prolonged or chronic fatigue, 1435 (7.7%) had prolonged fatigue, and 780 (4.2%) had chronic fatigue (seven cases refused to answer the fatigue questions). Among those 780 respondents with chronic fatigue, at Phase 1; 304 had ICF-like illness (e.g., not enough minor symptoms to be eligible for a CFS diagnosis), 68 had a CF-explained-like condition, and 408 had CFS-like profiles. All 408 members of the CFS-like group were invited to participate in Phase 2. Of this group of 408 individuals with CFS-like symptoms, the physician review team reviewed data on 166 individuals, who provided data during the Phase 2 evaluation. There were 47 individuals who were evaluated in a control group, and these individuals screened negative for CFS-like illness during Phase 1.

A team of four physicians and a psychiatrist were responsible for making a final diagnosis with two physicians independently rating each file using the current U.S. case definition of CFS [2]. Where physicians disagreed, a third physician rater was used [3]. Table 3 shows the number of cases in the control group (Control), individuals who were diagnosed with CFS using the Fukuda *et al.* [2] case definition (CFS), Idiopathic chronic fatigue (ICF, individuals who did not meet all the Fukuda criteria), and chronic fatigue explained (CF, *i.e.*, melancholic depression, bipolar disorders, anorexia nervosa/bulimia nervosa, psychotic disorders, drug or alcohol related disorders, or medical explanations for their fatigue).

**Table 3:** Community Epidemiology database *n* = 213.

Diagnosis	Percent Who Qualify for SEID
CFS (*n* = 32)	75% (*n* = 24)
ICF (*n* = 45)	44% (*n* = 20)
CF (*n* = 89)	47% (*n* = 42)
Control (*n* = 47)	6% (*n* = 3)

#### 2.3.3. SEID Diagnosis

To meet the SEID criteria [1] within this data set, a patient would need to have one of the following indications of 6 or more months of fatigue: fatigue for 6 or more months or fatigue present for more than 50% of the time for a minimum of 6 consecutive months. To meet substantial reduction from previous levels of functioning criteria, a patient needed to meet 2 of the following 3 criteria: role physical <50, social functioning <62.5, or vitality <35. To meet the post-exertional malaise criteria, a patient needed to report the occurrence of one of the following symptoms: prolonged generalized fatigue or malaise following previously tolerable levels of exercise, feeling generally worse than usual or fatigued for 24 h or more after exercise, or exercise brings on my fatigue. To meet the unrefreshing sleep criteria, a patient needed one of the following symptoms: after a night of sleep do you feel rested, after a night of sleep does your fatigue go away temporarily, needing to nap daily, problems falling/staying asleep. To meet the SEID criteria, the individual needed to meet either the cognitive impairment or orthostatic intolerance symptom. In order to meet the cognitive impairment criteria, a patient would need presence of at least one of the following cognitive items: forget recent conversations and events, confusion or distortion in familiar places, inability to concentrate, difficulty retaining information, only able to focus on one thing at a time, or new trouble with math. To meet the orthostatic intolerance criteria, a patient would need presence of at least one of the following items: sharp shooting pains in chest, rapid heartbeat, feeling unsteady on feet, often feeling dizzy, feeling weak or dizzy right after standing up.

#### 2.3.4. Estimating SEID Prevalence

Prevalence, which is the number to be estimated, is represented by P (p). The total number of respondents screened in Phase 1 (18,668) is N (N_t_). The proportion of screened positives (408/18,668 = 0.0219) is PI (π), and the proportion of screened negatives (18,260/18,668 = 0.9781) is 1 − PI (1 − π). The proportion of screened positives evaluated in Phase 2 who were diagnosed with SEID (Number of cases with SEID/166) is L1 (λ_1_), and the proportion of screened negatives evaluated in Phase 2 who were diagnosed with SEID (0/47 = 0.0) is L2 (λ_2_).

#### 2.3.5. Results

As indicated in Table 3, 75% (*n* = 24) of those in the CFS group met the SEID criteria, whereas 47% (*n* = 42) for the CF group, 44% (*n* = 20) for the ICF group, and 6% (*n* = 3) for the controls. Within the Chronic Fatigue explained by medical or psychiatric illness (CF), of those 19 with Melancholic Depression, 47% (*n* = 9) met the SEID criteria. In addition, for those with a medical reason for their fatigue, 48% (*n* = 16) met SEID criteria. In an effort to compare this new SEID case definition to the older Fukuda *et al.* [2] criteria, we computed the sensitivity and specificity. In this sample, the SEID criteria had a sensitivity of 0.75 and a specificity of 0.64.

This data set had been previously used to estimate the prevalence of CFS [3], which was 0.42. With the new number of SEID cases, we recalculated the prevalence rate, using methods described elsewhere [19]. This information was then used in the following formula to obtain the estimate of the prevalence [P = L1 × PI + L2 × (1 − PI) = L1 × 0.0219 + 0.0 × 0.9781]. The SEID Prevalence rate = L1 × PI + L2 × (1 − PI) = (89/166) × 0.0219 + 0.0 × 0.9781 = 0.0117. As the prior CFS Fukuda *et al.* [2] prevalence rate was 0.0042, the new SEID prevalence rate was 2.8 (0.0117/0.0042) times greater.

### 2.4. Study 4

#### 2.4.1. Procedure

We solicited participants with a diagnosis of MDD and CFS to participate in this study [20]. We administered to all participants the CDC Symptom Inventory, which assesses information about the presence, frequency, and intensity of 19 fatigue related symptoms during the past one month [21]. All eight of the critical Fukuda *et al.* [2] symptoms were included as well as 11 other symptoms (e.g., diarrhea, fever, sleeping problems, nausea, *etc.*). For each of the eight Fukuda *et al.* [2] symptoms, participants were asked to report the frequency (1 = a little of the time, 2 = some of the time, 3 = most of the time, 4 = all of the time) and severity (the ratings were transformed to the following scale: 0.08 = very mild, 1.6 = mild, 2.4 = moderate, 3.2 = severe, 4 = very severe). The frequency and severity scores were multiplied for each of the eight critical Fukuda *et al.* [2] symptoms and were then summed, in order to determine whether a person met the Fukuda *et al.* [2] criteria, as operationalized by Reeves *et al.* [22].

#### 2.4.2. Participants

We recruited 64 individuals, 27 with CFS and 37 with MDD. We obtained our sample of participants with CFS from two sources, local CFS support groups in Chicago and a previous research study conducted at DePaul University. To be included in the study, participants were required to have been diagnosed with CFS, using the Fukuda *et al.* [2] diagnostic criteria, by a certified physician and were required to currently meet CFS criteria using the Fukuda *et al.* criteria. We excluded individuals who had other current psychiatric conditions in addition to major depression or who reported having untreated medical illnesses (e.g., diabetes, anemia).

For the MDD group, we found participants from three sources, local chapters of the Depression and Bipolar Support Alliance group in Chicago; Craigslist—a free local classifieds ad forum that is community moderated; and online depression support groups. To be included in the study, all participants were required to have been diagnosed with a MDD by a licensed psychologist or psychiatrist. We excluded individuals who had other current psychiatric conditions in addition to a MDD (e.g., bipolar, schizophrenia) or who reported having untreated medical illnesses were also excluded. We carefully screened participants to ensure that participants from the MDD group did not have CFS as defined by the Fukuda *et al.* [2] criteria.

#### 2.4.3. SEID Diagnosis

To meet the SEID criteria [1] within this sample, a patient needed to have 6 or more months of illness. To meet substantial reduction from previous levels of functioning criteria, a patient needed to meet 2 of the following 3 criteria: role physical <50, social functioning <62.5, or vitality <35. To meet the post-exertional malaise criteria, a patient would need to have a frequency of at least some of the time and severity score of at least moderate to indicate prolonged levels of malaise following previously tolerated exercise. To meet the unrefreshing sleep criteria, patients would have to have indicated at least 1 of the unrefreshing sleep symptoms: Unrefreshing sleep in the past month, unrefreshing sleep present 6 months or longer, or trouble sleeping through falling or staying asleep. In order to meet the cognitive impairment criteria, a patient would need to have a frequency of at least some of the time and severity score of at least moderate to indicate impaired concentration. Due to a lack of items that tapped into orthostatic intolerance criteria, patients would instead need to meet the cognitive impairment criteria to qualify for this measure. In a prior study by Jason, Sunnquist, Kot, Brown, Newton *et al.* [14], when using the option to have orthostatic intolerance instead of cognitive impairment, only an additional 2% of participants meet the SEID criteria [14].

#### 2.4.4. Results

As indicated in Table 4, 81% (*n* = 22) of those in the CFS group met the SEID criteria, whereas 24% (*n* = 9) of those in the MDD group met SEID criteria. In an effort to compare this new SEID case definition to the older Fukuda *et al.* [2] criteria, we computed the sensitivity and specificity. The SEID criteria resulted in a sensitivity of 0.81 and a specificity of 0.76.

**Table 4:** CFS *vs.* MDD Database *n* = 64.

Diagnosis	Percent Who Qualify for SEID
CFS (*n* = 27)	81% (*n* = 22)
MDD (*n* = 37)	24% (*n* = 9)

## 3. Discussion and Conclusions

Table 1, Table 2, Table 3 and Table 4 indicate that the SEID criteria will probably select few individuals from healthy control samples, and although a few controls were identified as meeting SEID in Table 3, that control sample included a large group of individuals from the community, many of whom did have varying levels of fatigue and other problems. In addition, it appears that the SEID criteria do identify most of those who met the Fukuda criteria, as evidenced by the generally high sensitivity statistics; however, rates tend to be lower in Table 3, which is a community rather than tertiary sample, where symptom rates tend to be lower. Most importantly, the SEID criteria do tend to identify high rates of those with other medical illnesses, as indicated in Table 1 and Table 4 and the low specificity levels, and therefore many individuals with autoimmune and other health illnesses that had been exclusionary with prior case studies will now be comorbidity. In addition, as indicated in Table 2 and Table 4, many individuals with a purely affective disorder will now be also classified as having SEID.

Rates of SEID could increase due to the reduction of many exclusionary criteria. Based on study 3, using the Jason *et al.* [3] community-based epidemiologic study, 32 individuals had been classified as meeting the Fukuda *et al.* [2] criteria, for a prevalence rate of 0.42, but we estimate that 89 from this sample would now meet the SEID criteria, for a prevalence rate of 1.17, thus, the SEID prevalence rate would be 2.8 times as great. Of course, if our samples had only included those who had been selected patients had met the Fukuda *et al.* [2] criteria, as occurred in a recently published study [14], then those with many medical and psychiatric illnesses would have already been excluded, so in a study comprised of just those meeting the Fukuda *et al.* [2] criteria, the rates of those meeting the SEID criteria would be much more comparable to those meeting the CFS Fukuda criteria [14].

The current study suggests that the core SEID symptoms are not unique to SEID, as some patients with other illnesses, such as those evaluated in this study, have comparable symptoms. As a consequence, some patients with illnesses that had previously been exclusionary under past case definitions such as Fukuda *et al.* [2] will now be comorbid, possibly leading to an expanded number of individuals meeting SEID criteria. Even though the SEID criteria are for a clinical case definition [1], rather than a research case definition, it is likely that it will be used for research by investigators, as a similar process occurred with the clinical Fukuda *et al.* [2] CFS criteria. If there are ambiguities with case definitions, like what has occurred with the Fukuda *et al.* [2] CFS criteria, there will be difficulties in replicating findings across different laboratories, estimating the prevalence of the illness, consistently identifying biomarkers, and determining which treatments help patients. To develop or validate a reliable case definition, we need to both provide operationally explicit inclusionary and exclusionary criteria as well as develop a consensus within the scientific community for the case definition.

The current study suggests that some patients with MDD, who also have chronic fatigue, sleep disturbances, and poor concentration, will be misdiagnosed as having SEID. MDD can occur for anyone with a serious medical illness. Some patients might have been depressed prior to becoming ill with SEID, and probably others as a reaction to this illness [23]. However, patients with CFS have symptoms including night sweats, sore throats, and swollen lymph nodes, that are not commonly found in depression, and illness onset with CFS is often sudden, occurring over a few hours or days, whereas primary depression generally shows a more gradual onset [24,25]. Hawk, Jason, and Torres-Harding [15] were 100% successful in differentiating patients with CFS and MDD using the following variables: percent of time fatigue was reported, post-exertional malaise severity, unrefreshing sleep severity, confusion/disorientation severity, shortness of breath severity, and self-reproach.

Mood disorders are the most prevalent psychiatric disorders after anxiety disorders: for major depressive episode, the one-month prevalence is 2.2%, and lifetime prevalence is 5.8% [26]. The erroneous inclusion of people with primary psychiatric conditions in SEID samples would have detrimental consequences for the interpretation of epidemiologic, etiologic, and treatment efficacy findings for people with this illness. This is what occurred with another CFS case definition developed by the CDC [22]. Jason *et al.* [19] found that 38% of those with a diagnosis of a MDD were misclassified as having CFS using the CDC empirical case definition of Reeves *et al.* [22]. Fortunately, few adopted the Reeves *et al.* [22] empiric case definition, but the IOM [1] has considerably more prestige and influence, so their proposed SEID case definition criteria could ultimately have more far reaching effects. In study 3, 47% of those with Melancholic Depression met SEID criteria, whereas rates of MDD meeting SEID criteria in studies 2 and 4 were 27% and 24%, respectively. If individuals with primary affective disorder are misdiagnosed with SEID and provided cognitive behavioral treatment, they will more likely have positive outcomes, and this may create more difficulties in understanding the effects of these interventions for those who have ME (Myalgic Encephalomyelitis). Price, Mitchell, Tidy and Hunot [27] reviewed 15 studies of CBT with a total of 1043 participants with CFS. At treatment’s end, the CBT group showed more clinical improvement in contrast to those in usual care, but changes were not maintained at a one- to seven-month follow-up when including patients who had dropped out.

There are additional aspects of the IOM [1] case definition that have problems, besides exclusionary criteria. For example, it is unfortunate that there was a lack of a recommendation for a mental health evaluation, or a structured clinical interview, especially as some of these symptoms can overlap with primary affective or mood disorders. The SEID criteria require a patient to have either cognitive impairment or orthostatic illness, but orthostatic intolerance does not evidence prevalence rates as high as the other proposed core symptoms, whereas cognitive impairment does have higher prevalence rates [28]. Also, factor analytic studies do not support this system of a choice of cognitive impairment *vs.* orthostatic intolerance [29]. We believe this report did not adequately deal with the issue of whether distinct categories or continuous measures best capture patient differences, as there well might be different groupings of patients, with some having different features or more severity. Finally, empirical methods could have been employed to test the proposed classification system, and the committee members might have benefited from testing out their proposed model with an actual data set, as has recently been done [14].

There are a number of limitations in the present study. As we used archival data sets, some of the questions that have been proposed to define SEID were not available. Clearly, the current study needs to be replicated with questions that are now proposed [7], however, our questionnaires were able to assess that vast majority of issues and domains within SEID. In addition, several of our samples were relatively small, so larger studies are needed. Furthermore, we were only able to identify data sets representing a few illnesses, and more illnesses need to be investigated to assess whether some patients with these diagnoses might be included within the SEID classification system. It should be noted that samples recruited from patient organizations or that do not have an independent physician work up and diagnosis might be less reliable. The new SEID [1] criteria suggest frequency and severity ratings, many of which were not available from the data sets reported in the current study, so it is possible that some occurrence ratings selected less impaired individuals and inflated the number of patients meeting SEID criteria. Finally, none of the studies included a two-day exercise challenge, and such a test would be a better approach for documenting post-exertional malaise. However, such a test might exclude some of the individuals from a SEID diagnosis, and given that the SEID is a clinical criteria, most medical practitioners will not have access to this expensive two-day exercise test in the diagnostic process.

The recent IOM report [1] is being widely discussed among academics and the patient community [30]. There is a need to also consider how these recommendations will affect patients in other countries, given the prestige associated with an IOM report. The present study suggests that there might be a number of illnesses that had been exclusionary, which now might now be considered comorbid. This is a complex diagnostic decision, and there probably is a need for clearer rules regarding whether a person has an exclusionary or comorbid illness. Ultimately, we need investigations to help point to implications of using these new criteria, and ultimately, we need an open and inclusive process where all parties, including key gatekeepers including the patients, scientists, clinicians and government officials, are involved in the decision making process.
